# Examination of Genetic and Epigenetic Characteristics of Patients with Hyperhomocysteinemia Following High-Dose Folic Acid Consumption

**DOI:** 10.3390/nu17132133

**Published:** 2025-06-27

**Authors:** Barbara K. Bartak, Zsofia B. Nagy, Nikolett Szakallas, Alexandra Kalmar, Eszter Farkas, Fruzsina Banyai, Orsolya Pipek, Istvan Csabai, Nora Sydo, Emese Csulak, Bela Merkely, Istvan Takacs, Bela Molnar

**Affiliations:** 1 Department of Internal Medicine and Oncology, Semmelweis University, H-1083 Budapest, Hungary; molnar.barbara.kinga@semmelweis.hu (B.K.B.); nagy.zsofia@semmelweis.hu (Z.B.N.); kalmar.sopkez.alexandra@semmelweis.hu (A.K.); farkas.eszter.alexandra@semmelweis.hu (E.F.); banyai.fruzsina.ivett@semmelweis.hu (F.B.); takacs.istvan@semmelweis.hu (I.T.); 2 Department of Biological Physics, Eötvös Loránd University, H-1117 Budapest, Hungary; szakallasn3@student.elte.hu; 3 Department of Physics of Complex Systems, Eötvös Loránd University, H-1117 Budapest, Hungary; orsolya.pipek@ttk.elte.hu (O.P.); csabai@elte.hu (I.C.); 4 Heart and Vascular Centre, Faculty of Medicine, Semmelweis University, H-1122 Budapest, Hungary; sydo.nora@semmelweis.hu (N.S.); csulak.emese@semmelweis.hu (E.C.); rektor@semmelweis.hu (B.M.)

**Keywords:** hyperhomocysteinemia, folic acid, DNA methylation, biological age, whole-exome sequencing

## Abstract

**Purpose:** Homocysteine (HCY) metabolism is regulated by the methionine cycle, which is essential for DNA methylation and is associated with the folate cycle. This study examines the alterations in DNA methylation signature including epigenetic age changes, measure cell-free DNA (cfDNA), and HCY concentrations, and identifies genetic markers that may influence homocysteine response following folic acid (FA) supplementation in individuals with hyperhomocysteinemia (HHC). **Methods:** Blood samples were obtained from 43 HHC patients undergoing FA supplementation. We quantified FA and HCY levels, separated plasma and white blood cell fractions, and evaluated global DNA methylation using LINE-1 bisulfite pyrosequencing. Biological age was determined using Illumina BeadArray technology, and whole-exome sequencing was performed to investigate the patients’ genetic backgrounds. **Results:** Following FA supplementation, cfDNA levels significantly decreased and correlated positively with HCY (r = 0.2375). Elevated average LINE-1 methylation of cfDNA and PBMC-origin DNA was observed, with mean relative changes of 1.9% for both sample types. Regarding HCY levels, we categorized patients based on their response to FA supplementation. FA responders showed decreased HCY from 15.7 ± 5.5 to 11 ± 2.9 µmol/L, while in FA non-responders, an opposite trend was detected. The average biological age was reduced by 2.6 years, with a notable reduction observed in 80% of non-responders and 48% of responders. Sequencing identified mutations in several genes related to the one-carbon cycle, including *MTRR*, *CHAT*, and *MTHFD1*, with strong correlations to the non-responder phenotypes found in genes like *PRMT3*, *TYMS*, *DNMT3A*, and *HIF3A*. **Conclusions:** FA supplementation influences the HCY level, as well as affects the cfDNA amount and the DNA methylation pattern. However, genetic factors may play a crucial role in mediating individual responses to folate intake, emphasizing the need for personalized approaches in managing hyperhomocysteinemia.

## 1. Introduction

Sulfur-containing homocysteine (HCY) is a nonessential amino acid metabolite. It is produced through the methionine cycle in all cells and has a close relationship with the folate cycle, together forming the one-carbon metabolism. These processes are essential for various biochemical mechanisms, including epigenetic maintenance, nucleotide synthesis, and amino acid metabolism [1]. The biosynthesis of HCY begins with the absorption of dietary methionine; this is transformed into S-adenosylmethionine (SAM), which is the primary methyl donor molecule. After donating the methyl group for cellular methylation, SAM is converted into S-adenosylhomocysteine (SAH), which is finally converted to HCY, interacting with adenosine metabolism [2,3].

Folate, also known as vitamin B9, plays a crucial role in the remethylation of HCY back to methionine. This step is catalyzed by the enzyme methionine synthase (MS) and utilizes *N*-5-methyl tetrahydrofolate (THF) as the methyl group donor [2]. Folate serves as a cofactor that mediates the transfer of one-carbon units, not only for DNA methylation processes but also for the biosynthesis of purines and pyrimidines; thereby, it is involved in both genetic and epigenetic regulation [4,5,6]. The synthetic form of folate is known as folic acid (FA) [7]. It is more stable and is commonly found in nutritional supplements and fortified products [8]. FA supplementation can be an effective way to manage the homocysteine level [5,9]. However, there are known genetic factors that contribute to differences in individual responses to folate supplementation. One of the most extensively studied genes is methylenetetrahydrofolate reductase (*MTHFR*). This gene plays a crucial role in converting 5,10-methylenetetrahydrofolate into 5-methyltetrahydrofolate, which produces methyl donors for the conversion of homocysteine to methionine [10]. The *MTHFR* gene has been identified to possess two main mutations (C677T and A1298C), which are associated with moderate deficiency in its enzymatic activity, and thereby the decrease in HCY levels may also be reduced [11,12,13,14]. However, it is essential to identify additional genetic markers that may affect HCY response; exome sequencing could be an effective method for this purpose.

Generally, an HCY level higher than 15 μmol/L is defined as hyperhomocysteinemia (HHCY) [4,15]. HHCY is associated with several diseases, including psoriasis, type 2 diabetes mellitus, Alzheimer’s disease, vascular dementia, cardiovascular diseases, and different types of cancers [16,17,18,19]. Additionally, elevated HCY levels can enhance a process known as NETosis [20]. Neutrophil granulocytes actively eliminate pathogens through this process by releasing neutrophil extracellular traps (NETs), which consist of disintegrated chromatin and granular protein-bound chromatin [21]. While NETosis is a normal immune response aimed at eradicating pathogens, it can also contribute to host tissue damage associated with the numerous above-mentioned diseases. Since NETosis can be considered an active DNA secretion method, it can influence the amount of cell-free DNA (cfDNA) in the blood circulation. These observations suggest that the HCY level may have an impact on the cfDNA amount through the mechanism of NETosis [22,23].

Since folic acid is an important cofactor of the one-carbon cycle that actively modifies DNA methylation processes, it can influence the overall level of genome-wide global DNA methylation [24]. The methylation status of long-interspersed nuclear element 1 (LINE-1) retrotransposons represents this well, as they constitute approximately 17–25% of the human genome [25,26]. Furthermore, it can be measured easily and cost-effectively by using the pyrosequencing method. When the serum folate level is low, global hypomethylation can occur, leading to genome instability [27,28,29]. Therefore, FA supplementation may enhance the overall genome-wide methylation levels, potentially increasing the methylation of LINE-1 sequences. While LINE-1 methylation can be assessed in various cell types, the cfDNA fraction is also a suitable source for determining global DNA methylation [30,31,32].

Another approach for DNA methylation measurements is the bead-based microarray method. The Illumina bead array technology has seen significant advancements in recent years, as shown by the continuous increase in the number of analyzed CpG positions. The recently launched Infinium MethylationEPIC v2.0 (Illumina) DNA methylation profiling tool allows the simultaneous measurement of methylation levels at approximately 930,000 CpG sites across the entire human genome [33]. The previous versions of this method enable the development of epigenetic clocks suitable for measuring biological age, also known as “epigenetic age” or “DNA methylation age”. As discussed earlier, folic acid has a significant role in methylation regulation; therefore, we hypothesize that FA supplementation may influence the biological age of HHC patients.

These clocks for determining epigenetic age are based on the analysis of age-related methylation sites [34]. It has been shown that the age calculated from these sites is associated with several different diseases such as Parkinson’s disease, Alzheimer’s disease-related neuropathology, and different cancer types, and can predict overall mortality in humans [35,36]. One of the most well-known predictors of biological age was developed by Steve Horvath [37], who analyzed methylation data from various healthy and cancer tissues, and cancer cell lines using Illumina 27 K or Illumina 450 K array platforms. This method determines epigenetic age based on the methylation patterns of 353 selected CpG sites. Among these, 193 CpGs positively correlate and are hypermethylated with age, while 160 CpG positions negatively correlate and are hypomethylated with age. In the past decade, several generations of epigenetic clocks with various applications have been developed, including Hannum [38], Lee [39], PhenoAge [40], Zhang [41], and GrimAge [42]. The recent release of the next Illumina platform, called EPIC, has led to an extension of the Horvath clock’s scope to enhance its accuracy. This updated model is referred to as the “filtered H” model [43]. During our experiments we also generated EPIC data, and, therefore, we applied this model to accurately determine biological age in our analyses.

In the present study, as *MTHFR* gene mutations resulting in reduced enzyme activity are common in the Hungarian population [44], and since our mostly postmenopausal cohort might experience folate deficiency, we aimed to supplement high-dose folic acid to enhance absorption through passive diffusion [45], which becomes more prominent at elevated concentrations. Then we examined the effects of high-dose folic acid consumption on both genetic and epigenetic features in patients with hyperhomocysteinemia via serum homocysteine and plasma cfDNA levels. Our aims included the examination of the epigenetic pattern of plasma cfDNA and white blood cell-originated DNA, specifically focusing on changes in the LINE-1 methylation level and DNA methylation age. We categorized patients based on their response to FA supplementation regarding their homocysteine levels, and then we also analyzed their genetic background in conjunction with epigenetic factors. Our primary purpose was to demonstrate that while FA supplementation does not universally reduce the homocysteine level, it may have positive effects on other parameters.

## 2. Materials and Methods

### 2.1. Patients and Sample Collection

In total, 43 patients were enrolled in our study at the Department of Internal Medicine and Oncology, Semmelweis University, in Budapest, Hungary. All participants were diagnosed with hyperhomocysteinemia (>15 μmol/L). We excluded patients who had any current cancer, recent stroke or coronary event, and those receiving anti-folate treatment. The patients received high-dose folic acid (5 mg) daily for an average of four months. Fasting blood samples were taken at baseline and after the supplementation period, and were processed within 4 h of collection by double centrifugation at 1350 rcf for 12 min. Plasma fractions were then stored at −80 °C until use. Mononuclear cells and granulocytes were separated from the blood fraction using density gradient centrifugation with Histopaque-1119 and Histopaque-1077 (Sigma-Aldrich, Inc., St. Louis, MO, USA). In brief, 3 mL of Histopaque-1077 was layered over an equal volume of Histopaque-1119, and then 6 mL of whole blood was layered on top of the gradient. Centrifugation was performed at 700× *g* for 30 min at room temperature. The upper layer contained mononuclear cells, while granulocytes were found in the lower zone. The transferred cells were washed twice with 10 mL of Dulbecco′s Phosphate Buffered Saline (DPBS) (Sigma-Aldrich, Inc.) and then centrifuged for 10 min at 200 rcf. Finally, the cells were resuspended in 200 μL of DPBS solution and stored at −20 °C until needed. The study was carried out according to the Declaration of Helsinki and was approved by the local ethics committee and government authorities (National Centre for Public Health and Pharmacy, Budapest, Hungary; approval no. 2826-8/2023/EÜIG; approval date: 29 March 2023). Written informed consent was obtained from all patients before sample collection.

### 2.2. Folic Acid and Homocysteine Level Measurements

The amount of homocysteine was measured in all samples with enzyme cycling assay using a Roche Cobas C311 analyzer (Roche Diagnostics GmbH, Manheim, Germany) and the folic acid level was determined by immunoassay at the Department of Laboratory Medicine, Semmelweis University.

### 2.3. Cell-Free and Genomic DNA Isolation

Cell-free DNA (cfDNA) was extracted from 4 mL plasma samples using Quick-cfDNA Serum & Plasma Kit (Zymo Research, Irvine, CA, USA) following the manufacturer’s instructions. The cfDNA fraction was then eluted in 50 µL of elution buffer. To quantify the samples, a Qubit 1.0 fluorometer was used with the Qubit dsDNA High Sensitivity Assay Kit (Thermo Fisher Scientific, Waltham, MA, USA). Genomic DNA from white blood cells was isolated with the High Pure PCR Template Preparation Kit (Roche Diagnostics GmbH) following the Isolation of Nucleic Acids from Mammalian Whole Blood, Buffy Coat, or Cultured Cells protocol. This was supplemented with RNase A/T1 digestion (Thermo Fisher Scientific, Vilnius, Lithuania) for 1 h at 37 °C. Genomic DNA was eluted in 100 μL of elution buffer and stored at −20 °C until use. The concentration and purity of the samples were measured using a Qubit 1.0 fluorometer with the Qubit dsDNA HS Assay Kit (Thermo Fisher Scientific) as well as a NanoDrop-1000 spectrophotometer (Thermo Fisher Scientific).

### 2.4. Bisulfite Conversion

For pyrosequencing analyses, 300 ng of gDNA and the total amount of cfDNA were bisulfite converted using the EZ DNA Methylation Direct Kit (Zymo Research) following the manufacturer’s instructions, with an elution volume of 20 µL. For bead-based microarray measurements, 500 ng of granulocyte-derived gDNA underwent bisulfite conversion using the EZ DNA Methylation Kit (Zymo Research) according to the manufacturer’s protocol. The thermo-cycling conditions used were as follows: 16 cycles: 95 °C for 30 s, 50 °C for 1 h. The samples were eluted in 12 µL of elution buffer, and subsequent steps were performed immediately.

### 2.5. Bisulfite Pyrosequencing

One widely accepted technique for determining global DNA methylation is the bisulfite pyrosequencing of LINE-1 sequences. In brief, 30 ng of bisulfite converted genomic sample and the total amount of circulating DNA samples were processed to amplify a selected LINE-1 region with the PyroMark PCR Kit (Qiagen, Hilden, Germany). After completing the sample preparation steps, we used a PyroMark Q24 system (Qiagen) for pyrosequencing CpG LINE-1 assay (Qiagen, Cat no 970012) according to the PyroMark Q24 CpG LINE-1 Handbook (Qiagen). LINE-1 methylation was calculated by the PyroMark Q24 Software (Qiagen) at three selected CpG positions (331 to 318 of LINE-1; GenBank accession number: X58075) and the mean methylation percentages were determined. The relative change in LINE-1 methylation for each patient between the two sample collection points was calculated using the following formula: [100 × (after − before)/before].

### 2.6. Biological Age Determination

An Infinium MethylationEPIC BeadChip array (Illumina Inc., San Diego, CA, USA) was utilized to generate genome-wide methylation data from granulocyte-originated DNA samples. The bisulfite-treated DNA was processed using the Infinium HD Methylation Assay including hybridization to the Infinium MethylationEPIC v2.0 BeadChips according to the manufacturer’s protocol. To determine biological age, we utilized an epigenetic clock model based on the original multi-tissue Horvath clock [37], but adapted to the EPIC platform (“filtered H 272” model, as previously described by Pipek et al. [43]. Briefly, the model was trained on methylation data derived from diverse tissue types, incorporating measurements from multiple generations of Illumina methylation arrays (27 K, 450 K, and EPIC BeadChip platforms) to ensure broad applicability. From the original 354 CpG positions in the multi-tissue Horvath clock, 308 were retained for model building that were common across all three platforms. After retraining the coefficients, 272 CpG positions had non-zero weight in the final model. On EPIC array data, the model demonstrated superior performance compared to the original Horvath clock.

### 2.7. Whole-Exome Sequencing

The Shapiro–Wilk test was used to assess normality. For paired comparisons, either the Wilcoxon matched-pairs signed rank test or Student’s *t*-test was applied, depending on whether the data showed a non-normal or normal distribution, respectively, with a significance criterion of *p* < 0.05. The analyses were conducted using Prism8 software (GraphPad, San Diego, CA, USA).

During exome sequencing analysis, we focused on 41 genes, and among them, 29 genes are involved in the one-carbon metabolism cycle (Table S1) [46,47,48,49,50,51,52,53,54,55,56]. Moreover, we created a mutation database containing the annotated variants of the above genes by downloading and merging the corresponding data from ClinVar database.

Demultiplexing and FASTQ file generation were carried out using the Illumina BaseSpace interface. We employed the FastQC and MultiQC tools to assess the quality of sequencing reads. Raw sequence reads were aligned to the GRCh38 Human Reference Genome. SNP and short indel germline variants were called and determined by the Dragen Germline Variant Caller v.4.2.4 (Illumina Inc., San Diego, CA, USA). All the above variant analyses were performed using a .bed file containing the positions of the genes that are involved in the one-carbon cycle. These resulted in .vcf files of only those variants that were detected in the corresponding regions. Next, the generated variant calling (.vcf) files were imported to our server, which is used for data storage and evaluation. All the following data analyses were implemented on our local server computer. The .vcf files were annotated using the SnpEff eff variant annotation command line tool. Then the .vcf files were converted to .maf using the vcf2maf command line tool [57]. To visualize our general results, we used the plotmafsummary and oncoplot functions of the R maftools [58] package. Additionally, we completed a comprehensive statistical analysis using the IBM SPSS Statistics software (Version 30).

From this point onward, all steps were carried out using self-written Python v.3.13.3 scripts. First, we identified the genes that are mutated, both specifically and commonly. Next, we sorted the detected mutations into two categories: (1) annotated and classified, and (2) unknown and unclassified, based on their clinical significance using our ClinVar-derived mutation database. This analysis resulted in two sets of mutations, labeled as “classified” (detected mutations that meet the criteria for the first category) and “unclassified” (those that fall under the second category). All variants from both the responder and non-responder groups were stored and remain accessible. Through the classified variants, we were able to identify mutations that are specific to either the responder or non-responder group by listing alterations unique to each. We presented the mutated genes on graphs using the NetworkX Python package v.3.5 [59], highlighting their role in the one-carbon cycle. This created a map that allowed us to identify group-specific pathway hubs.

## 3. Results

### 3.1. Alterations of Folic Acid and Homocysteine Level

**Figure 1:**
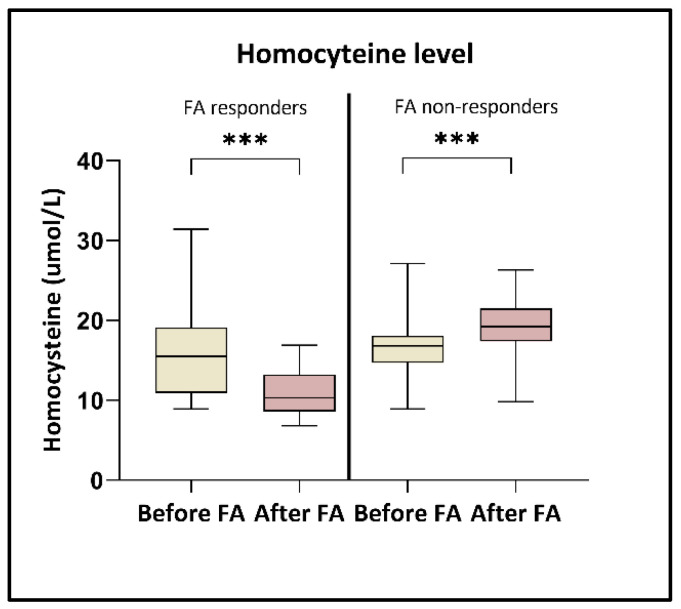
Homocysteine level alterations after folic acid supplementation. A significant (*** *p* < 0.0001) homocysteine reduction was detected in the FA responder group, while an elevated level (*** *p* < 0.0001) was observed in the FA non-responders.

The average folic acid amount was 46.3 ± 40 nmol/L at the beginning of the study, and after the high-dose folic acid supplementation, this level increased to 81.4 ± 29.4 nmol/L (*p* < 0.0001). In parallel, the HCY level was moderately decreased (from 16.3 ± 4.8 to 15.4 ± 5.5 µmol/L). However, after a closer inspection of the data, our measurements revealed two distinct groups regarding homocysteine responses (Figure 1). One group comprised patients (*n* = 21) who responded positively to the folic acid supplementation, resulting in decreased homocysteine levels (from 15.7 ± 5.5 to 11 ± 2.9 µmol/L) (*p* < 0.0001) (FA responders). In contrast, the other group included patients (*n* = 22) whose homocysteine levels remained unchanged or increased, rising from 16.7 ± 4.1 to 19.6 ± 3.7 µmol/L (*p* < 0.0001) (FA non-responders).

### 3.2. Cell-Free DNA Level Changes

**Figure 2:**
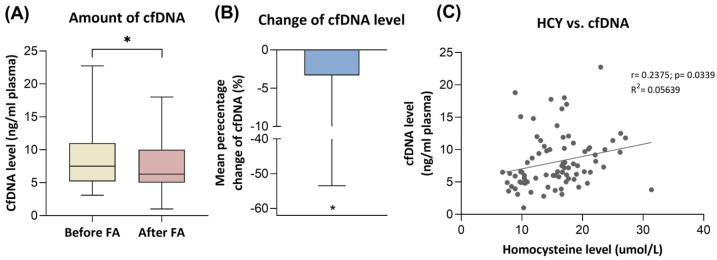
Changes in cfDNA levels and their relationship with homocysteine levels. (**A**) A significant decrease in cfDNA levels (* *p* < 0.05) was noticed following folic acid consumption. (**B**) The mean percentage change (%) of cfDNA from baseline to study end was −3.3% in the HHC patients. (**C**) A positive correlation was detected between homocysteine and cfDNA levels.

The level of cfDNA showed a slight but significant decrease (*p* < 0.05) in our patient group following folic acid supplementation, dropping from 9.0 ± 4.8 to 8.1 ± 4.9 ng/mL plasma (Figure 2). We did not observe significant differences between the groups when comparing FA responders and non-responders. The mean relative change from baseline to the end of the study was −3.3%. Correlation analysis revealed that the amount of cfDNA was positively correlated with the level of homocysteine (r = 0.2375; *p* < 0.05).

### 3.3. LINE-1 Methylation

**Figure 3:**
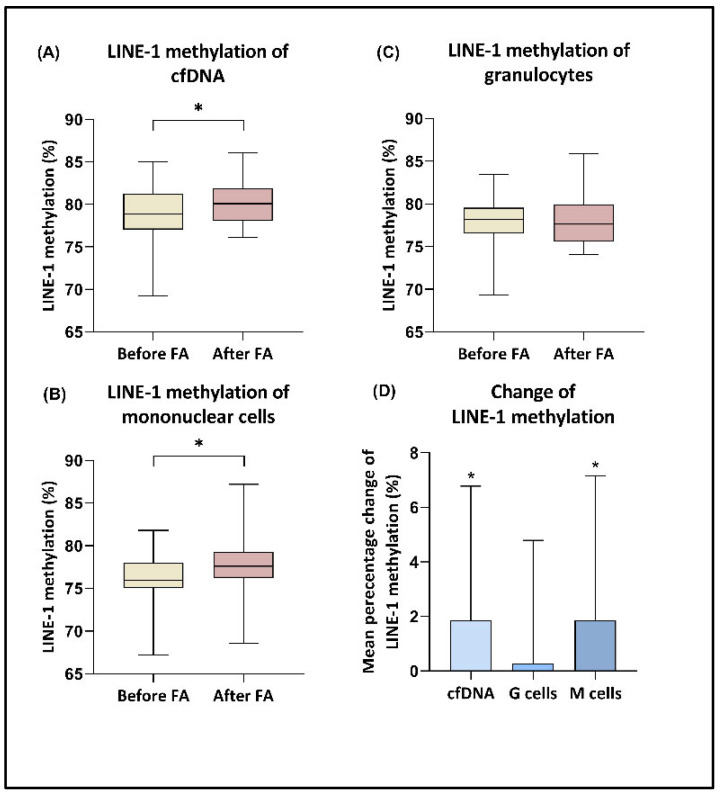
LINE-1 methylation levels in different sample types. (**A**,**B**) Elevated LINE-1 methylation levels were detected in cfDNA and mononuclear cell-origin DNA samples following the consumption of high-dose folic acid (* *p* < 0.05). (**C**) The LINE-1 methylation of granulocytes did not show significant alteration. (**D**) The mean percentage changes in LINE-1 methylation were significantly increased in the case of cfDNA (+1.9%) and M cells (+1.9%) (* *p* < 0.05).

The LINE-1 methylation levels of the cfDNA and gDNA fraction extracted from granulocytes and mononuclear cells were calculated by the average of the three analyzed CpG positions’ methylation. The detailed methylation percentages of the CpG sites measured at the beginning and end of the study are presented in Table 1. Elevated methylation levels of LINE-1 regions were observed in cfDNA and PBMC-origin DNA samples following the consumption of high-dose folic acid (*p* < 0.05), with mean relative changes in methylation of 1.9% for both sample types (Figure 3). In contrast, this increase was not observed in granulocyte fractions. Furthermore, if we examined the FA responder and non-responder groups separately, we found no significant difference in the values before and after FA supplementation. According to correlation analysis, a negative correlation was detected between HCY level and LINE-1 methylation of cfDNA and mononuclear cell-origin DNA in the FA responder group (r = −0.3866; *p* = 0.0239; r = −0.3508; *p* = 0.0265, respectively), while this connection was not observed in the non-responder patients (r = −0.03666, *p* = 0.8177; r = 0.1955, *p* = 0.2089, respectively).

### 3.4. Biological Age

**Figure 4:**
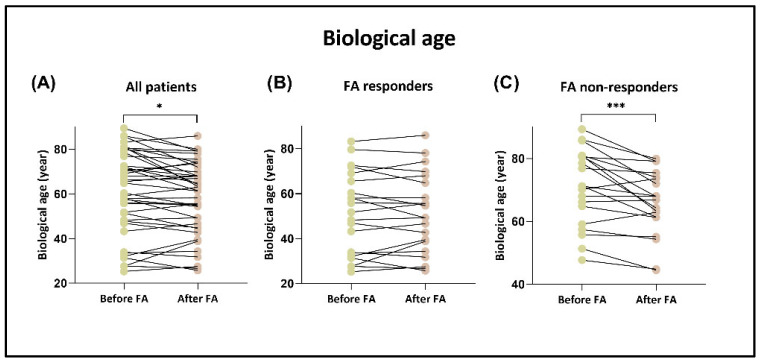
Changes in biological age following folic acid supplementation. (**A**) The DNA methylation age of the patients was significantly lower after folic acid consumption, with an average reduction of 2.6 years (* *p* < 0.05). (**B**,**C**) In patients grouped based on homocysteine response, the biological age was decreased by 0.5 years in FA responders, and by 5.3 years in FA non-responder patients (*** *p* < 0.001).

For biological age determination, we used a revised version of the original Horvath epigenetic clock (“filtered H 272” model; see Section 2.6). After folic acid consumption, the biological age of the patients was significantly decreased, by 2.6 years on average (*p* < 0.05) (Figure 4). Reduction was observed in 65% of the patients. We separately analyzed the changes in responder and non-responder groups based on homocysteine response. Interestingly, in the non-responder group, 80% of patients showed decreasing biological age after FA supplementation, by an average of 5.3 years (*p* < 0.001). In contrast, in the responder group, we detected a decline in only 48% of the individuals.

### 3.5. Exome Sequencing Results

During whole-exome sequencing, the analysis focused on genes that are crucial to one-carbon metabolism. First, we investigated the C677T and A1298C polymorphisms of the widely studied *MTHFR* gene. However, we did not find any differences between the patients categorized by their response to FA intake. C677T mutations were detected in heterozygous or homozygous form in 66.7% and 63.6% of FA responder and non-responder individuals. Similarly, A1298C mutations were observed in 38.1% and 40.9% of the groups, respectively.

**Figure 5:**
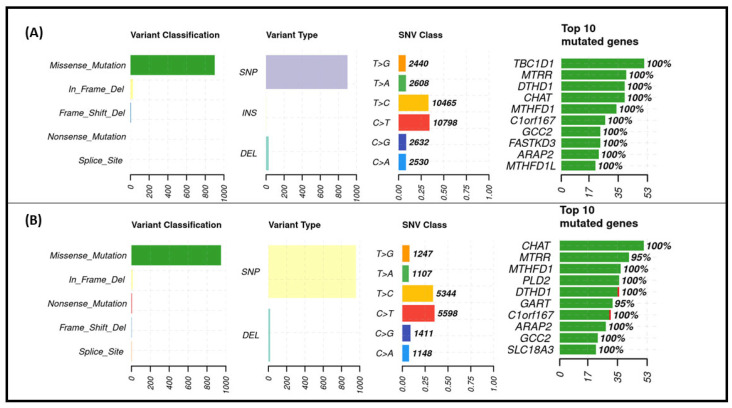
Mutation summary plots for two groups of patients based on their response to folic acid treatment: FA responders (**A**) and FA non-responders (**B**). Variant classification distribution: the X-axis represents the number of variants, and the Y-axis shows the variant type categories. Variant type plot: the X-axis indicates the number of variants, and the Y-axis displays both the variant type categories and the SNV class. Top 10 mutated genes: the X-axis shows the number of mutations, and the Y-axis lists the top 10 mutated genes.

A total of 41 genes were examined by exome sequencing, and their mutation profiles were determined in the patient sets. Altogether, 29 of these genes were closely linked to the one-carbon cycle (Table S1). Figure 5 shows the mutation summary plots of the selected DNA ranges for each group. Most of the identified variants were missense mutations observed in both the FA responder (A) and non-responder (B) groups. In FA responders, in-frame deletions and frame-shift deletions were the next most common variants, while in non-responders, in-frame deletions and nonsense mutations were predominant. Among the different types of variants, SNPs were the most common, followed by deletions, and the majority of the observed SNVs were of the types C > T, T > C, and C > G in both patient sets. The highly mutated genes among FA responders include *TBC1D1*, *MTRR*, and *DTHD1* genes; in contrast, in the non-responder groups, *CHAT*, *MTRR*, and *MTHFD1* were among the most mutated genes.

**Figure 6:**
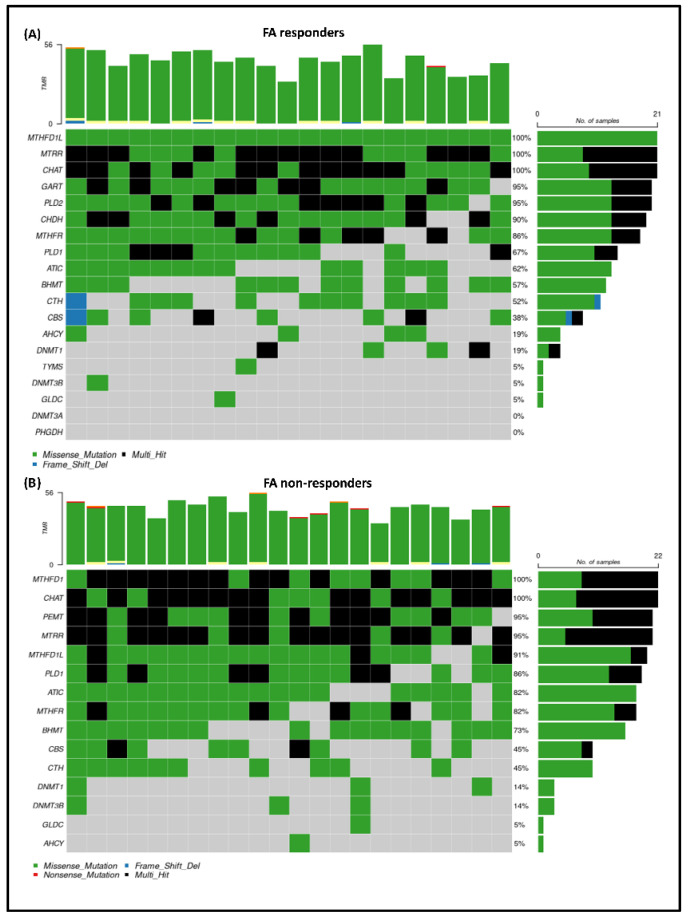
The distribution of gene mutations among the samples of the FA responder (**A**) and non-responder (**B**) group. The Y-axis lists the specific genes related to the one-carbon cycle, while the X-axis indicates the mutation rates. Different colors are used to represent various types of mutations: green for missense mutations, blue for frameshift deletions, red for nonsense mutations, and black for multi-hit mutations. A multi-hit mutation indicates that a gene has multiple mutations present in the corresponding sample. On the right side, a bar plot displays the exact mutation rates for the genes listed on the Y-axis. Additionally, the bar plot at the top shows the total number of mutations per sample.

To visualize the exact mutation rates of the genes belonging to the selected panel, we created oncoplots for the FA responder (Figure 6A) and FA non-responder (Figure 6B) groups separately. Our analysis revealed that the *MTHFD1L* gene exhibited the highest mutation rate among the FA responders, followed by the *MTRR*, *CHAT*, *GART*, and *PLD2* genes. In contrast, the mutation rate order for the non-responders was as follows: *MTHFD1*, *CHAT*, *PEMT*, *MTRR*, and *MTHFD1L*. This analysis indicates that while some genes are affected in both patient groups, specific differences were determined in the gene mutations between the two sets. Moreover, based on our results, we observed that certain genes carried multiple mutations, such as *MTRR* and *CHAT* genes.

**Figure 7:**
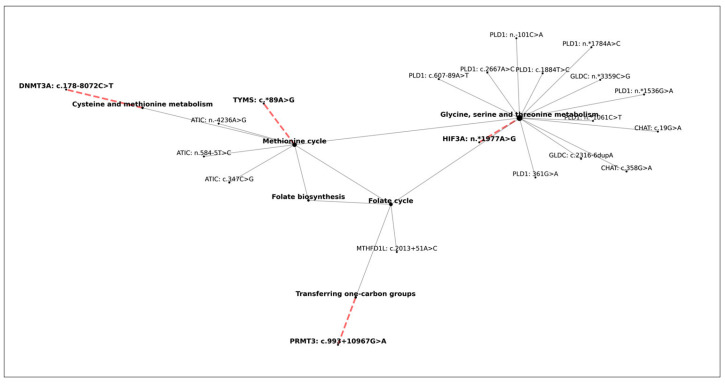
Graphical illustration of the hotspots of the FA non-responder group. The mutations occurring in at least 50% of the non-responder group can be seen in the figure. The main components of folic acid-related pathways are represented by central nodes, which correspond to pathways like transferring one-carbon groups, folate cycle, folate biosynthesis, methionine cycle, cysteine and methionine metabolism, and glycine, serine, and threonine metabolism. The surrounding nodes stand for the pathway-related gene-mutation pairs. In four cases, the central and surrounding nodes are connected by a red dashed line. These highlighted parts represent those pathway-related group-specific mutations that occur in at least 70% of FA non-responder patients, and were found in less than 50% of FA responders.

As a next step, we focused on the mutation hotspots of the FA non-responder group (Figure 7). The central nodes sign the folic acid-related pathways and the mutations that occur in at least 50% of FA non-responder patients, which can be seen in connection with these nodes. We identified four mutations (in *PRMT3*, *TYMS*, *DNMT3A*, and *HIF3A* genes) that occur in at least 70% of FA non-responder patients, but were found in less than 50% of FA responders. These mutations are marked with red dashed lines in Figure 7. The n.*1977A > G mutation of the *HIF3A* gene was found in 95.45% of non-responder patients (21/22), while in only 4.76% of FA responders (1/21). The analyzed mutations of *TYMS* and *DNMT3A* genes appeared in 81.82% (18/22) of the FA non-responder patient set, while the c.993 + 10967G > A mutation in the *PRMT3* gene appeared in 72.73% (16/22).

### 3.6. The Effect of One-Carbon Cycle Genes on Biological Aging

To characterize the effect of mutations in the one-carbon cycle-related genes, we carried out a linear regression and a Spearman rank order correlation analysis. The correlation matrix was constructed using the mutational data of all genes and the corresponding biological age differences per patient for the responder and also for the non-responder group. Our analysis revealed that there is no significant correlation between the number of mutations and the change in biological age in the case of responder patients. On the other hand, non-responder data showed that in the case of one gene, *CCT3*, the number of mutations positively correlates (r = 0.451, *p* = 0.035 < 0.05) with biological age difference after folic acid supplementation, although this result was not corrected for multiple testing. Based on the results of the linear regression analysis, we were able to describe the relation between the variables by a linear regression line: y = −9.214 + 0.394x, where x is the number of mutations in *CCT3* and y denotes the biological age difference (Table S2, Figure S1).

## 4. Discussion

Hyperhomocysteinemia is considered to be a clinical condition in which the blood homocysteine level exceeds 15 μmol/L [18,60,61,62,63]. There are several causes of high HCY levels, which are categorized into different groups such as genetic defects of enzymes, lack of cofactors involved in the metabolic pathways, excessive methionine intake, specific diseases, and use of certain drugs [64]. Essential cofactors of the one-carbon metabolism include vitamins such as B6, B12, choline, and folate [65], of which the most studied compound is folate. This is critical for the production of SAM, which is required for methylation processes. In the present study we examined the effects of high-dose folic acid consumption on both genetic and epigenetic features in patients with hyperhomocysteinemia.

According to our results, we observed that the level of homocysteine was moderately decreased in parallel with the elevation of folic acid (an average decrease of 3%). However, two clearly distinguishable groups were identified. The first group, referred to as FA responders, exhibited a significant average reduction in homocysteine levels of 27.2%. In contrast, the other portion of the patients did not show a lower HCY amount despite the increasing folic acid level (FA non-responders). In the background of the variations between individuals in the response of homocysteine level to folic acid supplementation may be different causes including gender, baseline HCY amount, smoking, or genetic factors such as mutations in *MTHFR* or cystathionine β-synthase (*CBS*) genes [66,67,68]. These genes have significant roles in the one-carbon metabolism. MTHFR protein converts 5,10-methylenetetrahydrofolate to 5-methyltetrahydrofolate, and a point mutation at position 677 of the *MTHFR* gene is linked to a milder enzymatic deficiency [12]. Meanwhile, the CBS gene encodes another essential enzyme that is involved in the transsulfuration of homocysteine produced during methyl group metabolism, with its deficiency leading to elevated plasma homocysteine levels [69]. Malinow et al. showed that patients homozygous for the T677 *MTHFR* allele indicated a larger reduction in plasma HCY levels after FA intake, while in homozygotes for the C677 allele, a moderately decreased HCY amount was detected [11]. Another study analyzed two polymorphisms located in the *CBS* gene, C699T and C1080T [70], and their results revealed that the *CBS* 699 and 1080 genotypes had significant effects on an individual’s response to HCY lowering by folic acid treatment. During our work, we conducted whole-exome sequencing to explore the genetic characteristics of the patients and identify specific alterations associated with the two differently responsive groups. Regarding *MTHFR* mutations, we also analyzed the C677T and A1298C polymorphisms; however, we did not find any differences in the occurrence of these mutations between the groups. This lack of variation may be attributed to the high prevalence of these mutations in the overall Hungarian population. Czeizel et al. investigated the geographical and ethnic variation of the C677T allele across 16 different areas worldwide, and according to their results the frequency of heterozygous condition was 45.2%, and the prevalence of homozygous genotype was 11.1% [44]. In our study, we observed comparable rates: the CT and TT genotypes were detected in 47.6% and 19.0% of participants, respectively, across both groups. Consequently, we shifted our focus to other mutations of the one-carbon cycle that occur in either the FA responder or non-responder patients. In the former group, the highest mutation rates were detected in *MTHFD1L*, *MTRR*, and *CHAT* genes, while in the non-responder patient set, *MTHFD1*, *CHAT*, and *PEMT* genes were the most mutated. One study analyzed a family with hereditary HHC, and its findings showed that the proband with severe thromboembolic disease who failed to accept folic acid therapy carried a homozygous variant of the *MTHFD1* gene, which did not occur in the sons who were sensitive to folic acid therapy [71]. Another study focused on the relationship between folate and choline intake, homocysteine metabolism, and genetic polymorphisms of several genes including *PEMT* in a cohort of healthy pregnant women [72]. Its results revealed that HCY concentrations varied significantly among different *PEMT* genotype subgroups, contingent upon the adequacy of choline and folate intake. Notably, carriers of the C allele who met their dietary needs for choline exhibited a 25% reduction in HCY levels, while those with sufficient folate intake showed a 20% decrease, in comparison to their counterparts who failed to meet these nutritional requirements. These observations indicate that specific mutations in one-carbon metabolism genes can affect individuals’ responses to folic acid treatment. Focusing on particular mutation hotspots of our FA non-responder group, we identified four mutations (*PRMT3* c.993 + 10967G > A, *TYMS* c.*89A > G, *DNMT3A* c.178072C > T, and *HIF3A* n.*1977A > G) that occurred in at least 70% of patients. Among these genes, *TYMS* and *DNMT3A* are directly part of the methionine cycle, which can regulate homocysteine levels. Additionally, the mutation of *HIF3A*—which also can be connected to the methionine cycle—could be identified in more than 95% of non-responder patients and only in one member of the responder group. To the best of our knowledge, this is the first study to link this *HIF3A* mutation with the homocysteine response to folic acid supplementation.

Our findings about the correlation between the mutations and DNA methylation levels indicate that the number of alterations in the *CCT3* gene has a moderate positive correlation with the observed biological age difference after folic acid supplementation.

It is known that *CCT3* plays a key role in cancer cell division, proliferation, metabolism, and drug resistance [73]; furthermore, as a part of the TRiC chaperonin complex [74], it is involved in telomere elongation maintenance by assisting the proper folding of the telomerase-associated protein TCAB1 [75]. The exact role of *CCT3* in biological aging requires further investigation.

Several studies have established a connection between changes in cell-free DNA and HCY levels [20,21,23,76]. In our previous work, we showed that these two parameters are positively correlated in colorectal cancer patients [23], and in individuals with progressive disease, the elevated HCY level is accompanied by an increased cfDNA level compared to patients who remained in remission after chemotherapy treatment. Another study indicated that an increased cfDNA amount is characteristic of the patients with essential hypertension and hyperhomocysteinemia in contrast to individuals without elevated HCY levels [20]. According to our findings, the level of cfDNA was significantly higher in the HHC patients prior to FA supplementation, and a reduction of 3.3% was observed as a result of FA intake. Additionally, we also detected a positive correlation between cfDNA and HCY level (r = 0.2375; *p* < 0.05). Li et al. demonstrated that neutrophil granulocytes of HHC patients can enhance the process of NETosis and showed which mechanism contributes to the elevation of cfDNA as an active cfDNA secretion method [20]. A study investigating patients with type 2 diabetes observed robust NETosis due to elevated HCY levels and increased components of NETs, including cfDNA, compared to healthy subjects [21]. These findings suggest that, in the case of HHC, the increase in cfDNA levels may result from an enhanced degree of NETosis.

Among the many functions of folic acid, it is an important cofactor of the DNA methylation process as it participates in the synthesis of SAM, the primary methyl donor molecule in the body. In our study, we investigated the relationship between folic acid supplementation and DNA methylation from various perspectives. To assess global DNA methylation levels, we applied pyrosequencing of LINE-1 sequences in cfDNA, as well as white blood cell-origin DNA fractions. Our measurements indicated a moderate, but significant, elevation of LINE-1 methylation in the sample types, with an average relative change of 1.9% observed in both cfDNA and PBMC DNA samples. These results suggest that FA intake may help maintain genome stability by increasing the methylation levels of LINE-1 sequences [77]. Additionally, we observed a significant negative correlation between homocysteine level and average LINE-1 methylation in these sample types of FA responder group (r = −0.3866; *p* = 0.0239; r = −0.3508; *p* = 0.0265, respectively), while this linkage was not detectable in the non-responder patients (r = −0.03666, *p* = 0.8177; r = 0.1955, *p* = 0.2089, respectively). The relationship between folic acid supplementation, homocysteine level, and LINE-1 methylation pattern is analyzed by several studies, but the results are quite contradictory. Fryer et al. conducted a study to analyze the connection between cord blood LINE-1 methylation and maternal folic acid intake, as well as cord blood folate and homocysteine levels in pregnant women [78]. They found that cord plasma homocysteine is inverse correlated with LINE-1 methylation (*p* = 0.001, r = −0.688), which is consistent with our results. Additionally, Pusceddu et al. observed that LINE-1 methylation at site 317 was elevated, with a mean percentage change of 0.81%, and the homocysteine level was decreased from 12 ± 4 nmol/L to 9 ± 3 nmol/L following 1-year supplementation of vitamin B12, B6, folate, vitamin D, and calcium [26]. Notably, these changes were not detected in individuals who were not supplemented with B vitamins. However, several other works have reported no significant correlations between these parameters. A study applied a supplementation of 500 μg folic acid, 500 μg vitamin B12, 50 mg vitamin B6, 1200 IE vitamin D, and 456 mg calcium for a period of one year [31]. The authors found a lower HCY level following the supplementation period, while the mean LINE-1 methylation and the LINE-1 methylation at the sites 305, 320, and 327 remained unchanged after 1 year. Pizzolo et al., similar to our study, examined the DNA methylation of mononuclear cells in patients with intermediate HHC [79]. They found that an eight-week daily intake of folic acid did not affect the methylation levels. Similar results were reported in another work, indicating that there were no changes in global DNA methylation in peripheral blood leukocytes of subjects with moderate HHC after three years of supplementation with 0.8 mg of folic acid [80]. There may be several factors contributing to the contradictory results observed. The amount of FA consumed and the duration of supplementation have a strong influence on the outcomes, as well as the baseline values of HCY and FA of the study groups. Moreover, as we mentioned earlier, the genetic background of the patients is also a very robust factor in their response to FA intake.

In addition to measuring the degree of LINE-1 methylation, we also applied another approach to examine the effect of FA consumption on epigenetic patterns. The Illumina bead array technology enabled us to simultaneously analyze the methylation level of numerous CpG positions across the human genome. According to our best knowledge, this is the first study that investigated the changes in the epigenetic age of HHC patients resulting from medium-term high-dose FA supplementation using bead array technology. Based on our results, the biological age was decreased in 65% of all the patients following the FA intake, and the rate of reduction was 2.6 years on average (*p* < 0.05). Sae-Lee et al. analyzed publicly available Illumina Infinium 450 K datasets from a work that supplemented 44 older patients with the combination of folic acid and vitamin B12 for 2 years [81]. They estimated the methylation age using Horvath’s epigenetic clock model [37] and analyzed the age acceleration residual by gender and MTHFR genotype. Their results showed that following vitamin intake, biological age is reduced in women with the *MTHFR* 677CC genotype. A study involving 16 healthy male volunteers examined the effects of daily folic acid supplementation at doses of either 400 μg or 800 μg over a period of 8 weeks [82]. They estimated the participants’ biological age using several epigenetic clocks, including Horvath’s DNAmAge. They calculated “AgeAccel” as the residual variation in each metric after adjusting for the participants’ chronological age at the time of sample collection and DNA methylation-based estimates of cell composition. The measurements indicated that FA supplementation positively influenced red blood cell and serum folate levels. However, the status of DNA methylation remained unchanged. Additionally, the study revealed only a modest correlation between the elevated folate levels and the rise in DNAmAge AgeAccel. During our experiments, we conducted a separate analysis of changes in FA responder and non-responder groups. Interestingly, in the non-responder group, 80% of patients experienced a decrease in biological age after FA supplementation, with an average reduction of 5.3 years (*p* < 0.001), while only 48% of the individuals in the responder group showed a lower biological age. These results suggest that a reduction in HCY levels is not necessarily linked to a decrease in biological age; rather, these two processes appear to be independent. However, the positive correlation between the number of mutations in gene *CCT3* and biological age change suggests that the alterations in specific genes can influence the positive or negative biological age tendency in non-responder patients.

The main limitation of the present study is the relatively small sample size, which may reduce statistical power, increase susceptibility to random errors, and limit the generalizability of the findings. On the other hand, the key strength is the relatively homogeneous Caucasian cohort, which reduces population-related variability.

Further follow-up of the current cohort offers the opportunity to investigate the long-term effects of folic acid supplementation. On these samples, we plan to conduct a long-read sequencing study using Oxford Nanopore Technology (ONT), which enables direct detection of DNA methylation and other base modifications without the need for bisulfite conversion or additional pre-sequencing treatments [83], giving us the potential to explore the landscape of modified bases after folic acid supplementation. These data will shed more light on the complex interplay between the observed mutations with DNA methylation and biological aging.

## 5. Conclusions

In our current study, we aimed to obtain a comprehensive picture of the responses of HHC patients to folic acid supplementation, including an analysis of genetic and epigenetic factors. We found correlations between the changes in homocysteine levels, cfDNA amounts, and global DNA methylation patterns. However, our results indicate that the response to FA consumption varies among patients, since among others factors, genetic background is a crucial influence.

## Figures and Tables

**Table 1:** LINE-1 methylation (%) as percentage of methylated CpG sites at baseline and after folic acid supplementation. The data are mean ± SD; statistically significant differences are in bold. * *p* < 0.05. av: average.

Samples Type	CpG Position	Baseline (%)	After FA Supplementation (%)
CfDNA	LINE-1 CpG1	85.0 ± 3.5	86.2 ± 3.7
	LINE-1 CpG2 *	**75.4 ± 4**	**77.2 ± 2.4**
	LINE-1 CpG3	75.9 ± 3.6	77.1 ± 3.6
	LINE-1 CpG av *	**78.8 ± 3**	**80.2 ± 2.5**
Granulocytes	LINE-1 CpG1	84.2 ± 3.4	84.4 ± 4.2
	LINE-1 CpG2	74.5 ± 2.5	74.8 ± 3.7
	LINE-1 CpG3	74.8 ± 3.8	74.5 ± 3.0
	LINE-1 CpG av	77.9 ± 2.6	78.1 ± 2.8
Mononuclear cells	LINE-1 CpG1	81.4 ± 3.8	82.1 ± 4.2
	LINE-1 CpG2	74.0 ± 2.7	74.5 ± 4.3
	LINE-1 CpG3 *	**73.5 ± 3.0**	**75.3 ± 3.7**
	LINE-1 CpG av *	**76.2 ± 2.7**	**77.5 ± 3.2**

## Data Availability

Due to privacy issues, we do not provide full data availability regarding the sequencing part of this publication. The data are used according to the consent provided by the participants without compromising their anonymity. Other relevant data are included in this article.

## Supplementary Materials

The following supporting information can be downloaded at: https://www.mdpi.com/article/10.3390/nu17132133/s1, Figure S1: Linear correlation analysis of the number of mutations in the *CCT3* gene with the observed biological age difference after folic acid supplementation; Table S1: Analyzed genes in the one-carbon cycle for exome genome sequencing; Table S2: Spearman rank correlation analysis of the number of CCT3 genes with the observed biological age difference after folic acid supplementation. References [46,47,48,49,50,51,52,53,54,55,56] are cited in the Supplementary Materials.
