# Supplementary material for: Examination of Genetic and Epigenetic Characteristics of Patients with Hyperhomocysteinemia Following High-Dose Folic Acid Consumption
Source: Nutrients. 2025 Jun 27;17(13):2133. doi: 10.3390/nu17132133 (PMC12251037; doi:10.3390/nu17132133)

Supplementary materials

Table S1. Analyzed genes in one-carbon cycle for exome genome sequencing.

Nr.	Gene ID	Gene name	Role	Part	References
1	AHCY	Adenosylhomocysteinase	Methionine degradation	Cysteine and methionine metabolism	[46]
2	ALDH1L2	Aldehyde Dehydrogenase 1 Family Member L2	One carbon pool by folate	Folate biosynthesis	[46]
3	ATIC	5-Aminoimidazole-4-Carboxamide Ribonucleotide Formyltransferase/IMP Cyclohydrolase	Antifolate resistance	Methionine cycle	[47]
4	BHMT	Betaine-Homocysteine S-Methyltransferase	Folate transport and metabolism	Cysteine and methionine metabolism	[48]
5	CBS	Cystathionine Beta-Synthase	Methionine degradation, Cysteine biosynthesis	Cysteine and methionine metabolism	[48]
6	CHAT	Choline O-Acetyltransferase	Glycerophospholipid metabolism	Glycine, serine and threonine metabolism	[49]
7	CHDH	Choline Dehydrogenase	Glycerophospholipid metabolism	Glycine, serine and threonine metabolism	[47]
8	CTH	Cystathionine Gamma-Lyase	Cysteine biosynthesis	Cysteine and methionine metabolism	[46]
9	DHFR	Dihydrofolate Reductase	Folate transport and metabolism, Antifolate resistance	Methionine cycle	[46,50]
10	DNMT1	DNA Methyltransferase 1	Methionine degradation	Cysteine and methionine metabolism	[51]
11	DNMT3A	DNA Methyltransferase 3 Alpha	Methionine degradation	Cysteine and methionine metabolism	[51]
12	DNMT3B	DNA Methyltransferase 3 Beta	Methionine degradation	Cysteine and methionine metabolism	[51]
13	GART	Phosphoribosylglycinamide Formyltransferase, Phosphoribosylglycinamide Synthetase, Phosphoribosylaminoimidazole Synthetase	Antifolate resistance	Methionine cycle	[52]
14	GLDC	Glycine Decarboxylase	Glycine cleavage system	Glycine, serine and threonine metabolism	[48]
15	HIF1A	Hypoxia Inducible Factor 1 Subunit Alpha	Choline metabolism in cancer	Glycine, serine and threonine metabolism	[53]
16	HIF3A	Hypoxia Inducible Factor 3 Subunit Alpha	Choline metabolism in cancer	Glycine, serine and threonine metabolism	[54]
17	MTHFD1	Methylenetetrahydrofolate Dehydrogenase 1	Folate transport and metabolism	Folate cycle	[46]
18	MTHFD1L	Monofunctional C1-Tetrahydrofolate Synthase, Mitochondrial	One carbon pool by folate	Folate cycle	[46]

19	MTHFR	Methylenetetrahydrofolate Reductase	Antifolate resistance	Methionine cycle	[48,50]
20	MTR	5-Methyltetrahydrofolate-Homocysteine Methyltransferase	Folate transport and metabolism	Cysteine and methionine metabolism	[50,52]
21	MTRR	5-Methyltetrahydrofolate-Homocysteine Methyltransferase Reductase	Cobalamin transport and metabolism, remethylation	Cysteine and methionine metabolism	[50,52]
22	PEMT	Phosphatidylethanolamine N-Methyltransferase	Glycerophospholipid metabolism	Glycine, serine and threonine metabolism	[51]
23	PHGDH	Phosphoglycerate Dehydrogenase	Glycine, serine and threonine metabolism	Cysteine and methionine metabolism	[47]
24	PLD1	Phospholipase D1	Glycerophospholipid metabolism	Glycine, serine and threonine metabolism	[55]
25	PLD2	Phospholipase D2	Glycerophospholipid metabolism	Glycine, serine and threonine metabolism	[55]
26	PRMT2	Protein Arginine Methyltransferase 2	type I protein arginine methyltransferase	Transferring one-carbon groups	[46,56]
27	PRMT3	Protein Arginine Methyltransferase 3	type I protein arginine methyltransferase	Transferring one-carbon groups	[46,56]
28	PRMT5	Protein Arginine Methyltransferase 5	type II protein arginine methyltransferase	Transferring one-carbon groups	[46,56]
29	TYMS	Thymidylate Synthetase	Folate transport and metabolism, Antifolate resistance	Methionine cycle	[46]

Table S2. Spearman Rank correlation analysis of the number of CCT3 gene with the observed biological age difference after folic acid supplementation.

Equation	Model Summary					Parameter Estimates	
	R Square	F	df1	df2	Sig.	Constant	b1
Linear	0.203	5.099	1	20	0.035	−9.214	0.394
The independent variable is CCT3							

Figure S1. Linear correlation analysis on the number of mutations in the *CCT3* gene with the observed biological age difference after folic acid supplementation.

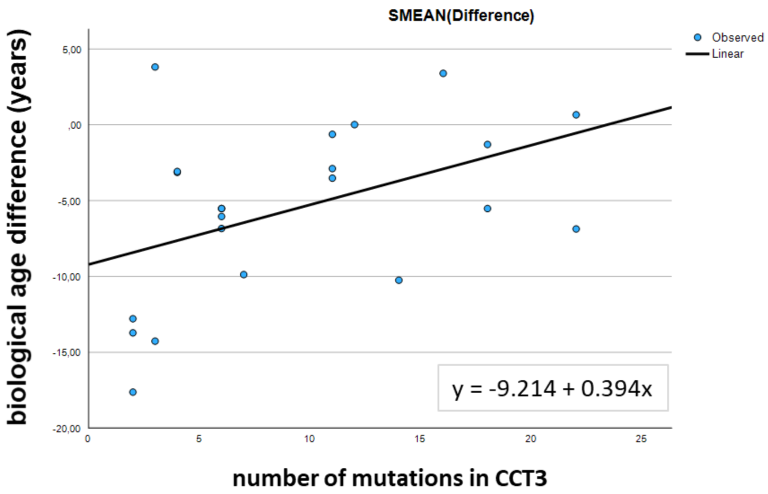

Supplement: Supplementary file 1 [file nutrients-17-02133-s001.zip › nutrients-3680541-supplementary.pdf]
